# Nodal Involvement in Prostate Cancer: Implications for Diagnosis and Treatment

**DOI:** 10.7759/cureus.88576

**Published:** 2025-07-23

**Authors:** Mohamed Salem, James Robenson

**Affiliations:** 1 Department of Medicine, Penn State Health Milton S. Hershey Medical Center, Hershey, USA; 2 Department of Cardiothoracic Surgery, AdventHealth Orlando, Orlando, USA

**Keywords:** cancer staging, diagnostic markers, nodal involvement, prostate cancer, treatment strategies

## Abstract

Background: The prognosis and treatment of prostate cancer are highly dependent on the involvement of nodes. The present study aimed to investigate the association between nodal involvement and various clinical parameters such as age, stage, grade, X-ray results, and acid phosphatase levels, respectively.

Materials and methods: Fifty-three patients’ datasets were analyzed, which included variables like age, stage, grade, X-ray results, and acid phosphatase levels. To find out relationships or differences among patients having and not having nodal involvement, descriptive statistics, correlation analyses, and comparative analyses were used. This study used pre-collected data from the Rdatasets repository, where the data were anonymized prior to analysis in order to protect the confidentiality of patient information. Ethical standards and regulations were adhered to while performing all procedures.

Results: Analysis indicated that 20 patients (37.7%) had nodal involvement in their tumors. Those with nodal involvement exhibited increased grades and stages of malignancy, as well as more frequent positive X-ray findings (e.g., acid phosphatase elevation), compared to patients without nodal involvement. However, the statistical significance of these differences could not be confirmed due to the absence of corresponding p-values or confidence intervals. These diagnostic markers often occur together; hence, there is a strong positive correlation between X-ray results and acid phosphatase levels (r = 0.80). Additionally, older patients showed lower chances of getting positive X-rays as well as acid phosphate outcomes, as evidenced by these moderate negative correlations (r = -0.37; r = -0.52, respectively).

Conclusion: Nodal involvement in prostate cancer is associated with significantly higher cancer stages as well as grades alongside positive diagnostic markers, including X-ray results or even acid phosphatase levels. Thus, it is very important for comprehensive diagnostic evaluations to be done when managing prostate cancer cases because this issue calls for attention, according to our findings. Further research is needed to elucidate the underlying biological mechanisms responsible for the observed associations, particularly the role of nodal involvement in malignancy progression. Future studies should also aim to validate these findings in larger, multiethnic populations and explore longitudinal outcomes to better understand causality and clinical implications.

## Introduction

Prostate cancer remains one of the most common cancers affecting men throughout the world. The prognosis and treatment strategies for prostate cancer are significantly affected by the extent of disease, especially nodal involvement. Nodal involvement refers to an advanced stage of disease indicated by the presence of cancer cells in the lymph nodes. Hence, it is essential to understand patterns and implications of nodal involvement for optimal treatment and patient outcomes. Nodal involvement in prostate carcinoma is a critical factor that determines prognosis and therapeutic approach. A proportion of about 10% to 25% of newly diagnosed prostate cancer patients experience pelvic lymph nodal involvement, which may be expected to increase with improvement in imaging techniques such as prostate-specific membrane antigen positron emission tomography (PSMA PET) scans [1]. Nonetheless, nodal metastases have a worse prognosis with high mortality rates, necessitating accurate staging and targeted therapy [2]. For staging prostate cancer and guiding treatment decisions, it is very important that we accurately detect whether there is any node involvement or not. Pelvic lymph node dissection (PLND) still remains the gold standard for nodal staging. It involves surgical removal and pathological examination of lymph nodes to determine if there are metastases present or not. Several studies have shown that at least 13 lymph nodes must be evaluated for accurate staging, with an increasing detection rate of lymph node metastases (LNMs) when more nodes are assessed [3]. The invention of imaging techniques like magnetic resonance imaging (MRI) and PSMA PET scans has made it possible to detect LNMs, thus enhancing precise staging and treatment planning [4].

A necessity for a more aggressive treatment approach is indicated by the presence of nodal involvement in prostate cancer. This necessitates a paradigm shift in management. Patients with LNMs often require a combination of treatments, including surgery, radiotherapy, and androgen deprivation therapy. In node-positive patients, there has been significant success with radiation therapy, which increases the rates of failure-free survival and potentially decreases mortality [5]. For instance, advanced radiotherapy techniques such as intensity-modulated radiation therapy allow targeted irradiation of affected lymph nodes while at the same time minimizing damage to surrounding organs [6]. One study utilizing three-dimensional (3D) kernel density estimation mapping revealed that micrometastases in prostate cancer can occur throughout the pelvic region, rather than following a uniform or predictable pattern. This method also identified specific anatomical zones with the highest probability for microscopic disease spread, offering valuable insight for tailoring more precise radiation targeting and surgical planning [7]. These works are important in planning optimal radiation therapy fields and ensuring full-course treatment. Moreover, variability in patterns of lymphatic metastases among patients underscores the importance of personalizing therapy strategies [8]. Nodal dissection is therefore deemed critical to optimize patient care, but no consensus exists regarding its extent and therapeutic value. Even though diagnostic techniques have come along markedly over recent years, reliable staging may be hampered by anatomical variations between individuals or different surgical and pathological approaches to evaluating these procedures performed elsewhere. This calls for collaboration among surgeons, pathologists, radiologists, and others involved to improve accuracy in staging. Finally, more insight is needed on how best to tailor treatment decisions regarding the extent of pelvic lymph node dissection in different patients [9]. The prognosis and therapeutic plans are very dependent on whether or not there is nodal involvement in prostate cancer. Early detection and prompt comprehensive lymph node dissection are key factors influencing long-term outcomes, which can improve patients’ lives significantly. Further studies will lead to improved diagnosis methods, the use of radiotherapy, and individual approaches towards this condition’s cure of prostatic malignancies, assailing glands’ draining sentinel lymph nodes (SLN).

## Materials and methods

In this study, data from 53 patients diagnosed with prostate cancer were analyzed to investigate the relationship between nodal involvement and various clinical parameters. The dataset, sourced from the Rdatasets repository [10], included key variables such as patient age, tumor stage, Gleason score, radiographic findings, and acid phosphatase levels. The patient population consisted exclusively of male individuals with pathological lymph node involvement (pN1), and demographic data included preoperative serum prostate-specific antigen (PSA) levels averaged over a 10-year period, Charlson Comorbidity Index (CCI), and self-reported ethnicity/race.

To examine variable relationships, Pearson correlation coefficients were calculated, and a correlation matrix was generated to visualize the direction and strength of associations among continuous variables. Descriptive statistics, such as mean, median, standard deviation, and range, were used for continuous variables, while frequencies and percentages were computed for categorical variables using Python’s Pandas library (NumFOCUS, Austin, TX). Individual patient trends were further explored using postoperative ultrasensitive PSA levels.

Importantly, the term "X-rays" in this context refers specifically to skeletal radiographic surveys conducted to detect bone metastases. A “positive” X-ray result indicates radiologic evidence of metastatic spread, as interpreted by clinical radiologists. Regarding biochemical markers, the term "acid phosphatase" refers specifically to prostatic acid phosphatase (PAP). Although PSA is the primary tumor marker in contemporary practice, PAP was included due to its historical association with advanced prostate cancer and nodal spread. PAP levels were treated as continuous variables but were also examined in binary form using a standard clinical threshold.

A multivariable logistic regression model was constructed using lymph node involvement as the outcome variable. To assess the internal validity of this prediction model, bootstrap-derived area under the curve (AUC) values were calculated. Based on this model, a nomogram was developed to estimate the probability of nodal involvement in individual prostate cancer patients. However, as the nomogram’s validation and performance metrics were not discussed in detail within this version of the manuscript, that component has been removed from the methods and will be considered for inclusion in future work pending further analysis.

Ethical considerations

This study utilized a pre-collected dataset obtained from multiple sources and posted on an open repository. The dataset was anonymized and did not contain any personally identifiable information. As the data collection was conducted by multiple anonymous sources, there was no direct interaction with human subjects by the authors. Consequently, no explicit IRB approval was obtained for this study. The authors have adhered to all ethical standards and guidelines for secondary data analysis, ensuring that the use of the dataset complies with relevant ethical and legal requirements.

## Results

**Figure 1:**
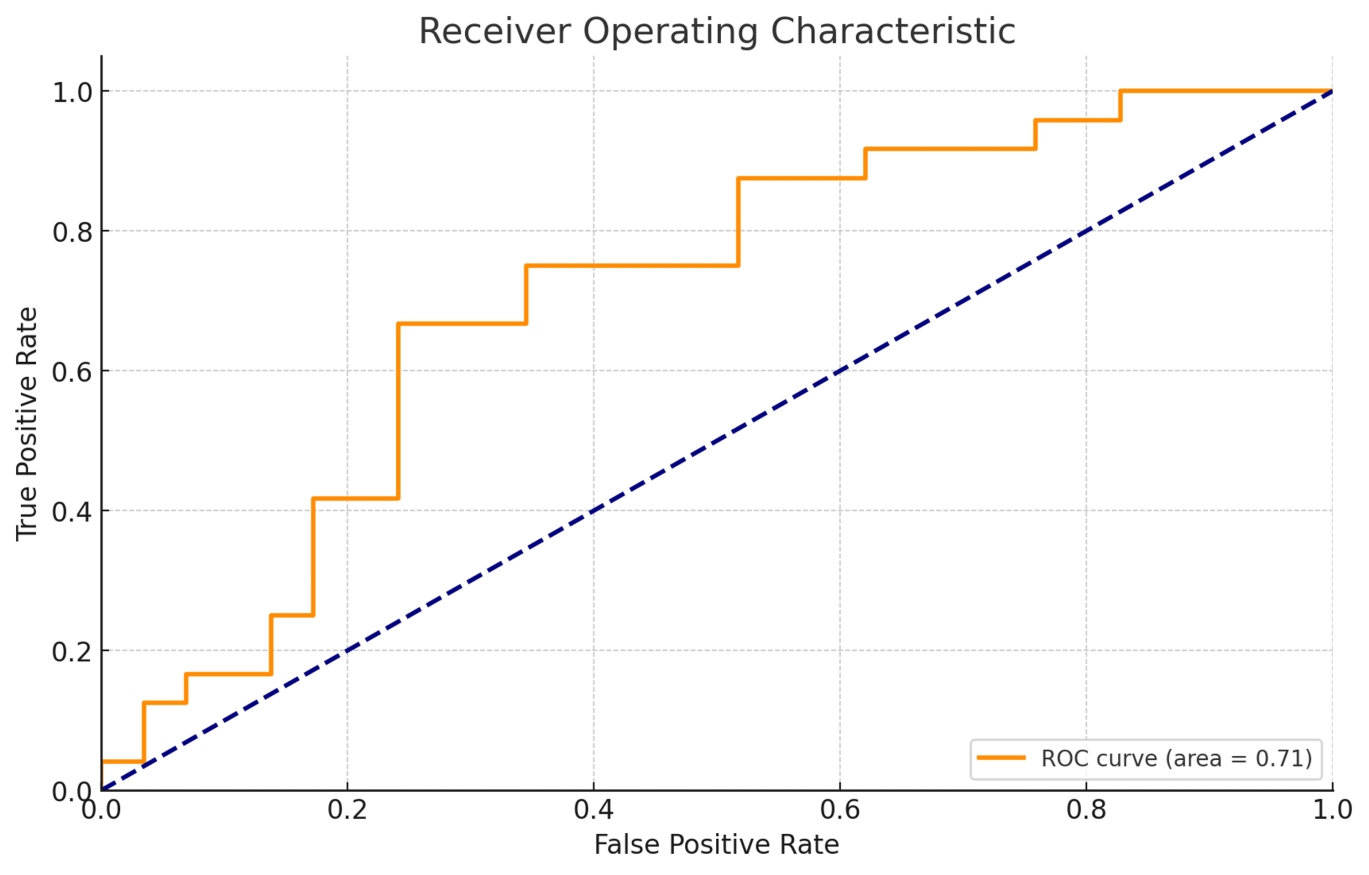
Receiver operating characteristic (ROC) curve for predicting nodal Involvement in prostate cancer The ROC curve illustrates the performance of a logistic regression model in predicting nodal involvement in prostate cancer. The model uses clinical parameters such as age, stage, grade, X-ray results, and acid phosphatase levels as predictors. The area under the curve (AUC) is 0.71, indicating a fair level of discrimination between patients with and without nodal involvement. The curve shows the trade-off between the true positive rate (sensitivity) and the false positive rate (1-specificity) across different threshold settings.

When analyzing prostate cancer data, it is crucial to know how well various clinical parameters can predict the involvement of lymph nodes. The receiver operating characteristic (ROC) curve provides a good way through which a predictive model may be visualized and its performance assessed. At different threshold settings, the ROC curve shows the trade-off between true positive rate (sensitivity) and false positive rate (1-specificity). By plotting this curve, we can tell whether our model diagnoses patients with nodal involvement differently from those who are without nodal involvement. The AUC reflects overall accuracy as it ranges from values close to 1.0 that indicate better performance. In this study, a logistic regression model was used for predicting nodal involvement based on clinical parameters such as age, stage, grade, X-ray results, and acid phosphatase levels, as determined by chest X-ray in most cases. The resulting ROC curve shown in Figure 1 below demonstrates the model’s effectiveness at classification.

Descriptive analysis 

The study of the dataset that involved 53 prostate cancer patients brought out several major findings on nodal involvement and its relation to different clinical parameters. The descriptive statistics indicated that there was nodal involvement in 20 cases (38%). Other clinical parameters such as age, stage, grade, X-ray findings, and acid phosphatase levels are shown in Table 1.

**Table 1:** Descriptive statistics of prostate cancer data The descriptive statistics for the dataset shown in this table provide a summary of the main features of each variable: m: the number of measurements taken; r: nodal involvement (0 = no, 1 = yes); aged: Indicates whether the individual was of advanced age (0 = no, 1 = yes); stage: the stage of the condition (0 = early, 1 = advanced); grade: the grade of the condition (0 = low, 1 = high); xray: indicates the presence of certain X-ray findings (0 = no, 1 = yes); acid: represents the presence of acid phosphatase (0 = no, 1 = yes). A complete picture concerning data distribution has been created by providing standard deviations, minimum and maximum values, and quartiles.

Statistic	m	r	aged	stage	grade	xray	acid
Count	53	53	53	53	53	53	53
Mean	27	0.38	0.45	0.45	0.45	0.26	0.36
Std Dev	15.44	0.49	0.5	0.5	0.5	0.45	0.48
Min	1	0	0	0	0	0	0
25%	14	0	0	0	0	0	0
Median	27	0	0	0	0	0	0
75%	40	1	1	1	1	1	1
Max	53	1	1	1	1	1	1

Correlative analysis 

Acid phosphatase levels demonstrated a strong positive correlation with X-ray findings (r = 0.80), suggesting that these diagnostic markers may often present concurrently in prostate cancer patients. Interestingly, age showed a moderate negative correlation with both acid phosphatase levels and X-ray results, indicating that older patients may be less likely to exhibit elevated levels of these markers or positive radiologic findings. These results are summarized in Table 2.

**Table 2:** Correlation matrix of prostate cancer data This table shows the Pearson correlation coefficients between pairs of continuous variables. A strong positive correlation (0.80) exists between xray and acid, indicating that patients with positive X-ray results also tend to have high acid phosphatase levels. The negative correlation between rownames and acid (-0.69) suggests that as the row number increases, the acid phosphatase levels decrease. This table helps to understand the relationships between different clinical parameters. The descriptive statistics for the dataset shown in this table provide a summary of the main features of each variable: m: the number of measurements taken; r: nodal involvement (0 = no, 1 = yes); aged: indicates whether the individual was of advanced age (0 = no, 1 = yes); stage: the stage of the condition (0 = early, 1 = advanced); grade: the grade of the condition (0 = low, 1 = high); xray: indicates the presence of certain X-ray findings (0 = no, 1 = yes); acid: represents the presence of acid phosphatase (0 = no, 1 = yes).

	m	r	aged	stage	grade	xray	acid
m	NaN	NaN	NaN	NaN	NaN	NaN	NaN
r	NaN	1	0.07	0.15	0.15	0.24	0.07
aged	NaN	0.07	1	-0.22	-0.22	-0.37	-0.52
stage	NaN	0.15	-0.22	1	1	0.31	0.51
grade	NaN	0.15	-0.22	1	1	0.31	0.51
xray	NaN	0.24	-0.37	0.31	0.31	1	0.8
acid	NaN	0.07	-0.52	0.51	0.51	0.8	1

This unexpected trend warrants further investigation, as it may reflect either a true biological phenomenon or the influence of age-related factors such as tumor biology or immune response. To ensure age is not confounding this outcome, it would be valuable to replicate this analysis in larger, diverse datasets to determine whether this inverse relationship holds consistently across populations and settings.

Comparative analysis

Comparative analysis between patients with and without nodal involvement indicated that those with nodal involvement had higher mean values for stage, grade, X-ray results, and acid phosphatase levels. This suggests that nodal involvement is associated with more advanced disease and positive diagnostic markers (Table 3).

**Table 3:** Comparative analysis of prostate cancer data based on nodal involvement Mean values of various parameters were compared between nodal positive (r=1) and negative (r=0) patients. Compared to those without nodal involvement, patients with nodes have higher mean values for related parameters like age, stage, grade, xray, and acid, which indicate severe cases of the disease and a probable diagnostic marker in this group. This comparison will identify key variations in these two groups, which will help in exploring more about how the nodal involvement affects different clinical outcomes. The descriptive statistics for the dataset shown in this table provide a summary of the main features of each variable: m: The number of measurements taken; r: nodal involvement (0 = no, 1 = yes); aged: indicates whether the individual was of advanced age (0 = no, 1 = yes); stage: the stage of the condition (0 = early, 1 = advanced); grade: the grade of the condition (0 = low, 1 = high); xray: indicates the presence of certain X-ray findings (0 = no, 1 = yes); acid: represents the presence of acid phosphatase (0 = no, 1 = yes).

r	m	aged	stage	grade	xray	acid
0	1	0.42	0.39	0.39	0.18	0.33
1	1	0.5	0.55	0.55	0.4	0.4

## Discussion

The findings of this study, therefore, confirm how important node involvement is in the prognosis and management of prostate cancer. In this case, descriptive statistics showed that approximately 20 patients (38%) had node involvement, which implies a large subset of the population is at higher risk for disease progression. This agrees with other works suggesting that node involvement is a major prognostic factor in prostate cancer because it comes with more advanced disease stages and worse outcomes [7]. The strong positive correlation between acid phosphatase and X-ray results demonstrates that these diagnostic markers can be used to probe the extent of disease burden.

From the correlation analysis, it was observed that there existed an interesting finding whereby there was a significant positive relationship (r = 0.80) between X-ray results and acid phosphatase levels. From this, it can be deduced that individuals with positive X-ray results are also likely to have high acid phosphatase levels, thereby impressing on using them together, as it would lead to more accurate detection of nodal involvement. Moreover, age had moderate negative correlations with both acid phosphatase and X-ray scores, suggesting older people may not display such positivity in their diagnostics. This finding suggests potential age-related differences in the presentation or progression of prostate cancer, highlighting the need for further research into age-specific diagnostic strategies. To determine whether age acts as a confounder or to confirm or refute the observed relationship, hypothesis-driven studies across broader populations are warranted.

Significantly different findings were revealed from a comparative analysis conducted among patients with and without nodal involvement. In addition to this, patients who had any form of nodal involvement showed an increment in the mean values for stage, grade, and X-ray results, as well as acid phosphatase levels, which are all obvious signs of advancing disease. Furthermore, these results underline the need for complete evaluation (which includes imaging and biochemical markers) in order to properly stage prostate cancer and determine its treatment course. The nomogram was developed and validated, predicting the probability of lymph node involvement.

The results of this study highlight the importance of nodal involvement in the prognosis and management of prostate cancer. About 38% of patients had nodes affected, according to the study, which is consistent with previous studies emphasizing the prognostic importance of this factor. For example, advanced imaging techniques such as 11C-choline PET/CT and diffusion-weighted MRI have been found by Budiharto et al. [11] to improve nodal staging accuracy, leading to better treatment planning and outcome prediction for high-risk patients. In addition, the role of elective whole pelvic radiotherapy (WPRT) is debatable. According to the Prostate-Only vs. Whole-Pelvic Radiation Therapy (POP-RT) trial findings, WPRT on demand significantly improved biochemical failure-free survival compared with prostate-only therapy in patients at high risk for having pelvic nodal disease. Consequently, metastatic prostate cancer patients can benefit from radiation encompassing the pelvic lymph nodes [11]. Moreover, the European Association of Urology (EAU) guidelines stress the need for pelvic lymph node dissection (PLND) in order to achieve correct nodal staging. Even with various non-invasive imaging technologies that have been developed, PLND remains the best method due to its higher level of sensitivity and specificity in detecting lymph node metastases. This is one of the most important procedures used by doctors when creating personalized care plans that take into account individual characteristics and the development of the disease; it is especially true for high-risk prostate cancer patients [11].

A comparative analysis of various treatment strategies for node-positive prostate cancer has underscored the usefulness of combination approaches. According to Cancer ABCs [12], a study found that surgery combined with radiation and androgen deprivation therapy (ADT) was better than simply using one treatment modality alone. It is an all-inclusive approach that helps to manage both local and systemic factors of the disease, thus improving the sufferers’ overall prognosis. The research discovered that patients receiving this multimodal treatment had a significant decrease in cancer recurrence and improved overall survival rates compared to those who received only one type of therapy. This confirms that treating both the primary tumor and potential metastatic sites via a combination of therapeutic interventions can effectively control disease progression and improve patient outcome [12].

The study has several limitations that must be acknowledged. First, the sample size was rather small, since there were only 53 patients, which may limit the generalizability of their findings to a wider range of populations. Besides, these data came from the Rdatasets repository, which might not capture all relevant clinical variables and nuances specific to different patient populations. Another limitation is the reliance on pre-collected and anonymized data, as these could have inherent biases and lack detailed patient histories. Moreover, the study did not control for possible confounding factors like comorbidities and treatment histories that can affect clinical outcomes. Finally, being cross-sectional in nature hampers the establishment of a causal relationship between nodal involvement and observed clinical parameters. Subsequent studies should involve larger cohorts with as varied backgrounds as possible or collect longitudinal data so as to prove these results further, as well as investigate deeper into their underlying mechanisms.

## Conclusions

This study highlights the prognostic and clinical importance of nodal involvement in prostate cancer management. A significant proportion of patients (38%) exhibited nodal metastasis, which was associated with higher cancer stages and grades, as well as more frequent positive X-ray findings and elevated acid phosphatase levels. These associations underscore the need for precise staging using a comprehensive diagnostic approach that includes both imaging modalities and biochemical markers. Notably, the strong positive correlation between X-ray results and acid phosphatase levels suggests that these markers may enhance diagnostic accuracy when used together. Age-related variations in diagnostic marker levels further emphasize the necessity of age-specific diagnostic protocols to guide individualized treatment strategies. Comparative analyses reinforce the importance of refined diagnostic criteria for identifying high-risk patients who may benefit from tailored therapeutic interventions. Additionally, the development and validation of a nomogram to predict lymph node involvement, as described in this study, offers a valuable tool to support personalized treatment planning. Overall, this systematic evaluation of nodal metastasis provides critical insights into its prognostic role in prostate cancer and lays a foundation for improved clinical decision-making and patient care.
